# Serum Amyloid P Aids Complement-Mediated Immunity to Streptococcus pneumoniae

**DOI:** 10.1371/journal.ppat.0030120

**Published:** 2007-09-07

**Authors:** Jose Yuste, Marina Botto, Stephen E Bottoms, Jeremy S Brown

**Affiliations:** 1 Centre for Respiratory Research, Department of Medicine, Royal Free and University College Medical School, Rayne Institute, London, United Kingdom; 2 Molecular Genetics and Rheumatology Section, Faculty of Medicine, Imperial College, London, United Kingdom; University of Pennsylvania, United States of America

## Abstract

The physiological functions of the acute phase protein serum amyloid P (SAP) component are not well defined, although they are likely to be important, as no natural state of SAP deficiency has been reported. We have investigated the role of SAP for innate immunity to the important human pathogen Streptococcus pneumoniae. Using flow cytometry assays, we show that SAP binds to S. pneumoniae, increases classical pathway–dependent deposition of complement on the bacteria, and improves the efficiency of phagocytosis. As a consequence, in mouse models of infection, mice genetically engineered to be SAP-deficient had an impaired early inflammatory response to S. pneumoniae pneumonia and were unable to control bacterial replication, leading to the rapid development of fatal infection. Complement deposition, phagocytosis, and control of S. pneumoniae pneumonia were all improved by complementation with human SAP. These results demonstrate a novel and physiologically significant role for SAP for complement-mediated immunity against an important bacterial pathogen, and provide further evidence for the importance of the classical complement pathway for innate immunity.

## Author Summary

Serum amyloid P (SAP) is a protein that is found in high concentrations in the blood, the exact function(s) of which are not clear. However, no known natural state of SAP deficiency has been identified, which suggests that SAP does have a vital role in human health. SAP can bind to molecular patterns found on the surface of bacteria, and it has been proposed that this may mark bacteria for attack by the immune system. We have investigated whether SAP helps protect against an important bacterial pathogen, Streptococcus pneumoniae. We show that SAP binds to different strains of S. pneumoniae, and that this leads to activation of an important component of the immune response called the complement system. Complement is particularly important for defence against S. pneumoniae infections, and using animal models of infection, we demonstrate that loss of SAP makes mice more susceptible to S. pneumoniae pneumonia. These results suggest that SAP helps the immune system to recognise invasion by bacteria and describe a new mechanism required for control of S. pneumoniae infections. This study may help the design of new therapeutic strategies to prevent or treat important bacterial diseases.

## Introduction

The pentraxin serum amyloid P (SAP) is a glycoprotein that is a major constituent of human serum, present in concentrations of about 30–50 μg/ml. Pentraxin proteins are distinguished by their pentameric assembly and calcium-dependent ligand binding, and include another important serum protein, C reactive protein (CRP). Pentraxins are components of the acute phase response, with serum levels of SAP increasing markedly in mice during sepsis [1,2]. No known natural state of SAP deficiency has been identified, suggesting that SAP has a vital role in human health, but the exact function(s) of SAP are ill-defined. SAP binds to DNA, chromatin, and apoptotic cells, and is thought to aid their clearance [3,4]. However, by binding to amyloid fibrils and stabilising their structure, SAP also promotes amyloid persistence and is therefore an important component of the disease amyloidosis [5–7]. Interactions of SAP with the immune system have also been described, the physiological relevance of which is not clear. These include binding to the complement factor C1q and preventing the inhibitory function of the complement regulatory component C4 binding protein (C4BP), both of which can lead to activation of the classical pathway of complement [8–11], and improving Fcγ receptor–mediated phagocytosis of zymosan and apoptotic cells [12–15]. In addition, SAP can bind to structures found on microbial surfaces, including lipopolysaccharide (LPS), phosphorylcholine (PC), and terminal mannose or galactose glycan residues [16–20]. As a consequence, SAP can bind to a range of microbes, including Gram-positive and Gram-negative bacterial pathogens and human influenza A virus [21,22].

These data suggest that SAP might act as a pathogen recognition receptor and assist innate immunity to microbial pathogens, which is analogous to the known role of CRP, to which SAP has 51% homology at the amino acid level [19,23]. Several studies have investigated the potential role of SAP for host immunity, but have produced conflicting results. SAP enhances killing of Listeria monocytogenes by macrophages without affecting phagocytosis [24], inhibits the growth of intra-erythrocyte malaria parasites [25], reduces uptake of Mycobacterium tuberculosis by macrophages [26,27], and prevents influenza A infection of cell cultures [22], phenotypes which could improve immunity to these pathogens. However, data from experimental infections in SAP-deficient mice have shown that SAP has little effect on immunity to influenza A [28]. Furthermore, SAP prevents classical pathway complement deposition and phagocytosis of rough strains of Escherichia coli, and SAP-deficient mice are protected against infection with rough strains of E. coli and Streptococcus pyogenes [29,30]. In contrast, SAP does not bind to or affect phagocytosis of a smooth strain of E. coli, and SAP-deficient mice had increased susceptibility to this E. coli strain by unknown mechanisms [29,30]. At present, whether SAP aids immunity to a common human pathogen has not been clearly demonstrated.

One human pathogen of major importance worldwide is *Streptococcus pneumoniae. S. pneumoniae* is the second most common cause of death due to bacterial infection, is responsible for the majority of cases of pneumonia, and is a significant cause of septicaemia and meningitis in both infants and adults [31–33]. The high mortality of severe S. pneumoniae infections (over 20% even if treated with appropriate antibiotics) and the spread of antibiotic resistance amongst clinical strains underline the importance of understanding host immunity to S. pneumoniae. Experimental and human data have convincingly demonstrated the essential role of the complement system for preventing S. pneumoniae infections and for controlling replication of S. pneumoniae within the lungs and the systemic circulation [34–36]. We have previously reported that, in contrast to S. pyogenes, even in the absence of specific acquired antibody the classical pathway is the most important complement pathway for innate immunity to S. pneumoniae [34]. The mechanisms by which the classical pathway is activated by S. pneumoniae infection include recognition of PC on the bacterial surface by natural IgM and CRP [34,37–40], and binding of bacterial capsular polysaccharide to the lectin SIGN-R1 expressed on marginal zone macrophages within the spleen [41]. As SAP also binds to PC and is known to interact with the classical pathway, we hypothesised that SAP could be an additional mediator of classical pathway activity against S. pneumoniae and contribute towards innate immunity to this important pathogen. In this study, we investigated the role of SAP for immunity to S. pneumoniae, in particular its role during complement activation, phagocytosis, and infection in mouse models of disease using mice genetically engineered to be deficient in SAP.

## Results

### SAP Binds to Different Capsular Serotypes of S. pneumoniae

**Figure 1:**
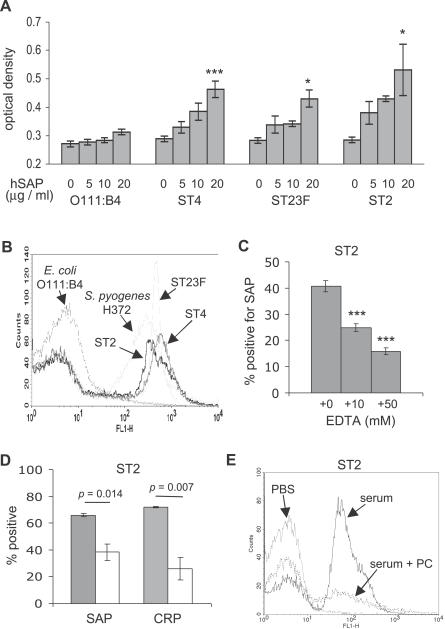
Binding of hSAP to ST2, ST4, and ST23F Strains of S. pneumoniae (A) Results of whole-cell ELISAs presented as maximum ODs after incubation with different concentrations of hSAP (given below each column as μg/ml), using the E. coli O111:B4 strain as a negative control. Error bars represent SDs, and asterisks mark significant *p*-values for comparisons of results for S. pneumoniae in 20 μg/ml hSAP versus medium alone (2-tailed *t* tests, *p < 0.01, ***p < 0.0001). (B) Examples of flow cytometry histograms demonstrating hSAP binding to the surface of the three different S. pneumoniae strains, the E. coli O111:B4 strain, and the S. pyogenes H372 strain (positive control) after incubation in human serum. (C) Effect of different concentrations of EDTA on SAP binding to the ST2 S. pneumoniae strain in human serum. Error bars represent SDs, and asterisks mark significant *p*-values for comparisons of results for EDTA versus serum alone (2-tailed *t* tests, ***p < 0.0001). (D) Effect of addition of 100 mM PC on SAP and CRP binding to the ST2 S. pneumoniae strain in human serum. Grey columns, results for human serum; white columns, results for human serum in the presence of PC. Addition of 100 mM bovine serum albumin had no effect on SAP binding (unpublished data). Error bars represent SDs, and *p*-values are indicated above the columns. (E) Examples of flow cytometry histograms demonstrating inhibition of hSAP binding to the surface of the ST2 S. pneumoniae strains by addition of 100 mM PC to human serum.

Whole-cell ELISAs were used to investigate whether human SAP (hSAP) binds to three S. pneumoniae strains representing capsular serotypes (STs) 2, 23F, and 4 using the smooth E. coli O111:B4 strain, which is known not to bind to SAP [30], as a negative control. Dose-dependent binding to purified hSAP was demonstrated to all three S. pneumoniae strains (Figure 1A). To confirm these results for live bacteria in a physiological medium, hSAP binding to S. pneumoniae incubated in human serum was assessed by a flow cytometry assay using the S. pyogenes H372 strain and the E. coli O111:B4 as positive and negative controls, respectively. For all three S. pneumoniae strains and S. pyogenes, a significant proportion of bacteria were positive for hSAP (ST2 38% standard deviation [SD] 10, ST4 32% SD 8.3, ST23F 39% SD 12, H372 50% SD 2.2), whereas the E. coli O111:B4 strain showed no significant binding to hSAP (6.7% SD 4.0) (Figure 1B). Addition of EDTA reduced the proportion of the ST2 S. pneumoniae strain positive for SAP (Figure 1C), demonstrating that SAP binding was calcium dependent. Furthermore, the presence of PC inhibited binding of both SAP and CRP (used as a positive control, as CRP is known to bind to S. pneumoniae cell wall PC) to a similar degree, suggesting that PC is a major ligand for SAP binding to S. pneumoniae (Figure 1D and 1E). Significant levels of SAP binding still occurred in the presence of PC or high concentrations of EDTA, and this might reflect some non-specific binding of SAP to S. pneumoniae. These data show that SAP can bind to a range of S. pneumoniae strains, and therefore could potentially act as a pathogen recognition receptor and mediate innate immune responses to this pathogen.

### C3b Deposition on S. pneumoniae Is Partially Dependent on SAP

**Figure 2:**
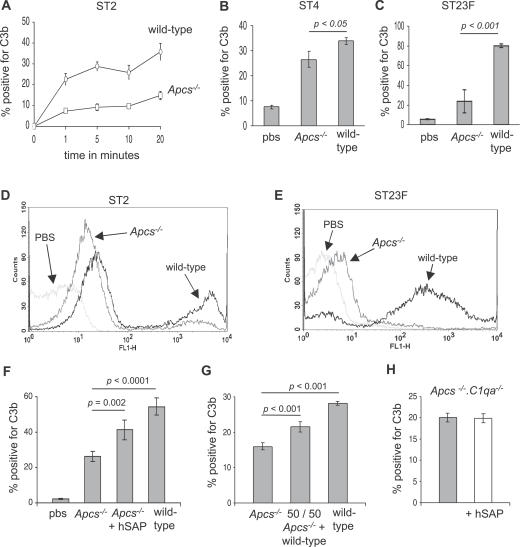
Effects of SAP on C3b Deposition on S. pneumoniae Measured Using Flow Cytometry (A) Time course of the proportion of ST2 S. pneumoniae bacteria positive for C3b after incubation in serum from wild-type and *Apcs^−/−^* mice. For the comparison of results for wild-type versus *Apcs^−/−^* mice, *p* < 0.001 at all time points from 1 to 20 min. (B and C) Proportion of ST4 (B) and ST23F (C) S. pneumoniae bacteria positive for C3b after incubation for 20 min in serum from wild-type and *Apcs^−/−^* mice. (D and E) Examples of flow cytometry histograms of C3b deposition on ST2 (D) and ST23F (E) S. pneumoniae strains after incubation in PBS or serum from wild-type or *Apcs^−/−^* mice. (F) Effect on the proportion of ST2 S. pneumoniae positive for C3b of addition of 10 μg/ml hSAP to serum from *Apcs^−/−^* mice. (G) Effect on the proportion of ST2 S. pneumoniae positive for C3b of addition of an equal volume of serum from wild-type mice to serum from *Apcs^−/−^* mice. (H) Effect of addition of hSAP (50 μg/ml, white column) on C3b deposition on the ST2 S. pneumoniae strain in serum from *Apcs* ^−/−^ *.C1qa* ^−/−^ mice. Grey columns, results for *Apcs* ^−/−^ *.C1qa* ^−/−^ serum; white columns, results for *Apcs* ^−/−^ *.C1qa* ^−/−^ serum in the presence of hSAP. For panels (A–C) and (F–H), error bars represent SDs, and in (A) when not visible are too small to be seen outside of the symbol. *p*-Values are calculated using 2-tailed *t* tests.

To determine whether SAP affects complement activation by S. pneumoniae, C3b deposition on S. pneumoniae incubated in serum from wild-type or mice genetically engineered to be deficient in SAP (*Apcs^−/−^* mice) was analysed using a flow cytometry assay. Starting 1 min after incubation in serum, C3b deposition on the ST2 S. pneumoniae strain was strongly impaired in serum from *Apcs^−/−^* mice at all time points compared to the results for serum from wild-type mice (Figure 2A and 2D). The proportion of bacteria positive for C3b after incubation in serum from *Apcs^−/−^* mice was also reduced for the ST4 and ST23F strains (Figure 2B, 2C, and 2E). The effect of SAP deficiency on C3b deposition varied between the three S. pneumoniae strains investigated, with a very marked effect for the ST23F strain, a relatively weak effect for the ST4 strain, and an intermediate effect on the ST2 strain. Backcrossing of SAP-deficient *Apcs^−/−^* animals onto the C57BL/6 genetic background results in the translocation of surrounding chromosomal DNA from the mouse 129 strain into the C57BL/6 strain, and this combination has been shown to explain some of the phenotypes associated with *Apcs^−/−^* mice [12]. However, C3b deposition on the ST2 strain in serum from congenic C57BL/6 mice engineered to carry a similar 129 fragment but no deletion of the SAP gene (C57BL/6.129(D1Mit105–223)) [42] was identical to C3b deposition in serum from wild-type C57BL/6 mice (unpublished data), demonstrating that this genetic combination was not responsible for the impaired C3b deposition detected in serum from *Apcs^−/−^* mice. Furthermore, C3 deposition on the ST2 strain in *Apcs^−/−^* serum was partially restored by addition of serum from wild-type mice and by the addition of exogenous hSAP (Figure 2F and 2G). These results confirm that the reduced complement deposition on S. pneumoniae in serum from *Apcs^−/−^* mice is due to loss of SAP, and suggest that hSAP has a similar functional effect on complement activation by S. pneumoniae as mouse SAP.

### SAP Increases C3b Deposition on S. pneumoniae through the Classical Pathway

**Figure 3:**
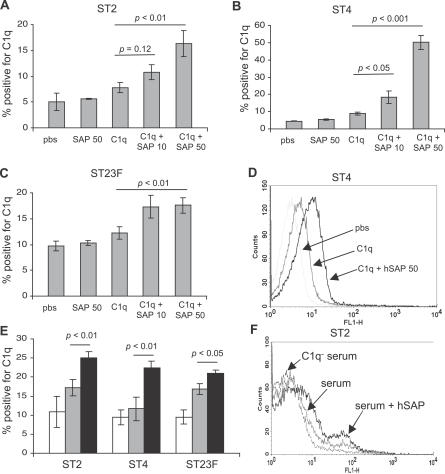
Effects of SAP on C1q Binding to S. pneumoniae Measured Using Flow Cytometry (A–C) Proportion of ST2 (A), ST4 (B), and ST23F (C) S. pneumoniae bacteria positive for C1q after incubation in 90 μg/ml human C1q with or without addition of 10 or 50 μg/ml hSAP. (D) Example of flow cytometry histograms of C1q binding to ST4 S. pneumoniae after incubation in 90 μg/ml human C1q with or without addition of 50 μg/ml hSAP. (E) Proportion of S. pneumoniae positive for C1q after incubation in human serum with (black shading) or without (grey shading) addition of 50 μg/ml hSAP, using C1q-depleted serum (white shading) as a negative control. (F) Example of flow cytometry histograms of C1q binding to ST2 S. pneumoniae after incubation in human serum with or without addition of 50 μg/ml hSAP. For panels (A–C) and (E), error bars represent SDs, and *p*-values are calculated using 2-tailed *t* tests.

As SAP has been shown to bind to C1q [9], the first component of the classical pathway, one mechanism by which the SAP may aid C3b deposition on S. pneumoniae could involve increasing C1q binding to the bacteria and thereby activating the classical pathway. To investigate this possibility, the three serotypes of S. pneumoniae were incubated with physiological concentrations of purified human C1q protein and hSAP protein, and the deposition of C1q on the bacteria analysed using a flow cytometry assay. For all three strains, the presence of hSAP increased the deposition of hC1q on the S. pneumoniae surface (Figure 3A–3D). Furthermore, addition of exogenous hSAP to human sera increased C1q deposition on all three S. pneumoniae serotypes (Figure 3E and 3F). To confirm that the effects of SAP on C3b deposition are mainly mediated by increased C1q deposition activating the classical pathway, C3b deposition assays were repeated using serum from mice with deficiencies of both SAP and C1q (*Apcs* ^−/−^.*C1qa* ^−/−^) with and without addition of exogenous hSAP. In contrast to the results for *Apcs^−/−^* serum, addition of hSAP to *Apcs* ^−/−^.*C1qa* ^−/−^ serum had no effect on C3b deposition on the ST2 S. pneumoniae strain (Figure 2H). These data suggest that SAP-mediated C3b deposition on S. pneumoniae is C1q and therefore classical pathway dependent.

### Impaired Phagocytosis of S. pneumoniae in Serum from *Apcs^−/−^* Mice

**Figure 4:**
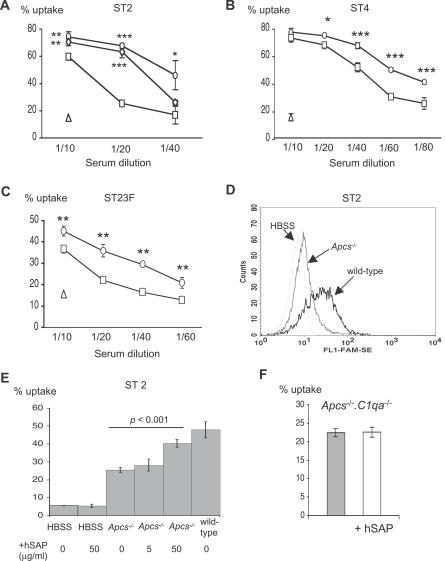
Effect of SAP on Phagocytosis of S. pneumoniae (A–C) Phagocytosis (presented as proportion of HL60 cells associated with fluorescent bacteria) of (A) ST2, (B) ST4, and (C) ST23F after incubation in different dilutions of serum from wild-type (circles) or *Apcs^−/−^* (squares) mice. Results for incubation in HBSS are shown by the triangle symbol, and for the ST2 strain the results for a 50:50 mix of serum from wild-type and *Apcs^−/−^* mice (diamonds) are also included. (D) Example of a flow cytometry histogram of phagocytosis of ST2 S. pneumoniae by HL60 cells after incubation in HBSS or serum from wild-type or *Apcs^−/−^* mice. (E) Effect of addition of 5 or 50 μg/ml exogenous hSAP on phagocytosis of the ST2 S. pneumoniae strain in serum from *Apcs^−/−^* mice. (F) Effect of addition of hSAP (50 μg/ml) on phagocytosis of the ST2 S. pneumoniae strain in serum from *Apcs* ^−/−^ *.C1qa* ^−/−^ mice. Grey column, results for *Apcs* ^−/−^ *.C1qa* ^−/−^ serum; white column, results for *Apcs* ^−/−^ *.C1qa* ^−/−^ serum in the presence of hSAP. For panels (A–C), (E), and (F), asterisks mark significant *p*-values for comparisons of results for wild-type or mixed serum to *Apcs^−/−^* serum (2-tailed *t* tests, *p < 0.01, **p < 0.001, ***p < 0.0001). All error bars represent SDs and when not visible are too small to be seen outside the symbol.

To study the functional consequences of reduced complement deposition on *S. pneumoniae* in *Apcs^−/−^* serum, we analysed phagocytosis of S. pneumoniae in serum from *Apcs^−/−^* mice using flow cytometry to assess the proportion of fluorescent bacteria associated with HL60 cells, a human neutrophil cell line. In this assay, the association of fluorescent bacteria with phagocytes is mainly due to phagocytosis of the bacteria rather than simple binding to the cell surface [43]. Phagocytosis of all three S. pneumoniae strains was mainly serum dependent, with only low levels of uptake after incubation in Hank's Balanced Salt Solution (HBSS) medium alone (Figure 4). Phagocytosis was consistently reduced when the bacteria were incubated with serum from *Apcs^−/−^* mice compared to serum from wild-type mice for the three S. pneumoniae strains investigated (Figure 4). The level of phagocytosis was partially restored in serum from *Apcs^−/−^* mice when mixed with serum from wild-type mice or by addition of exogenous hSAP (Figure 4A and 4E). However, addition of hSAP to HBSS alone or to *Apcs* ^−/−^.*C1qa* ^−/−^ serum did not stimulate phagocytosis of S. pneumoniae, suggesting that the effects of hSAP are dependent on the classical pathway (Figure 4E and 4F). These results are consistent with the results of the C3b deposition assays, and indicate that reduced opsonisation of S. pneumoniae with C3b in *Apcs^−/−^* serum is associated with a reduced efficiency of phagocytosis.

### Clearance of S. pneumoniae from the Blood Is Impaired in SAP-Deficient Mice

**Figure 5:**
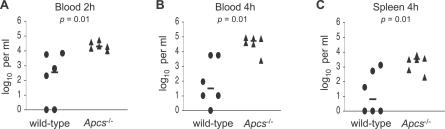
Clearance of the ST2 S. pneumoniae Strain from Wild-Type and *Apcs* ^−/−^ Mice Inoculated Intravenously with 1.0 × 10^6^ cfu Each data point represents log_10_ cfu/ml results for a single mouse, with the bar showing the median for each group. (A) Results for blood 2 h after inoculation. (B) Results for blood 4 h after inoculation. (C) Results for spleen homogenates 4 h after inoculation. Data is obtained from one experiment that is representative of two separate experiments. *p*-Values for Mann–Whitney *U* comparisons between wild-type and *Apcs* ^−/−^ mice are given below the title for each panel.

Clearance of S. pneumoniae from the circulation is thought to be dependent on complement and on phagocytosis by the reticuloendothelial system [44]. To test whether the effects of SAP deficiency on complement deposition and phagocytosis of S. pneumoniae result in an impaired ability to clear bacteria from the blood, wild-type and *Apcs^−/−^* mice were inoculated by i.v. injection with 2 × 10^5^ cfu of the ST2 strain D39, and bacterial cfu in the blood calculated by serial dilutions at 2 and 4 h post-inoculation and in spleen homogenates 4 h post-inoculation (Figure 5). *Apcs^−/−^* mice had between 2 and 3 logs greater cfu in both the blood and spleen compared to wild-type mice, demonstrating that *Apcs^−/−^* mice have a marked impairment in their ability to clear S. pneumoniae from the systemic circulation consistent with the impaired phagocytosis of S. pneumoniae found in SAP-deficient serum.

### SAP-Deficient Mice Are Susceptible to S. pneumoniae Pneumonia

**Figure 6:**
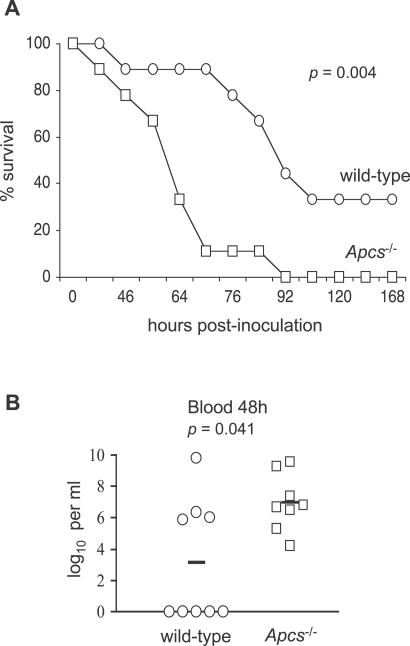
Susceptibility of *Apcs* ^−/−^ Mice to S. pneumoniae Pneumonia (A) Progression to lethal disease in groups of nine wild-type (circles) and *Apcs* ^−/−^ (squares) mice inoculated i.n. with 1.0 × 10^6^ cfu of ST2 S. pneumoniae. For the comparison between wild-type and *Apcs^−/−^* mice, *p* = 0.004 (log rank test). (B) S. pneumoniae cfu in blood recovered from wild-type (circles) and *Apcs^−/−^* (squares) mice 48 h after i.n. with 1.0 × 10^6^ cfu of ST2 *S. pneumoniae.* Bars represent the median cfu recovered for each group, and the *p*-value for the comparison between wild-type and *Apcs* ^−/−^ mice was 0.041 (Mann–Whitney *U* test).

A mouse model of pneumonia was used to confirm a biological role of SAP for innate immunity to S. pneumoniae. C57BL/6 mice are partially susceptible to the ST2 S. pneumoniae strain D39 after intranasal (i.n.) inoculation and therefore provide a sensitive model for identifying immunological defects that result in increased susceptibility [34]. Groups of wild-type and *Apcs^−/−^* C57BL/6 mice were inoculated i.n. (to mimic the natural route of infection) with 1 × 10^6^ cfu of D39 of S. pneumoniae and the development of lethal infection monitored. Lethal disease developed faster in *Apcs^−/−^* mice, with a median time to fatal infection of 64 h (interquartile range [IQR] 52 to 68 h) compared to 86 h for wild-type mice (IQR 77 to 92 h), and all the *Apcs^−/−^* mice developed lethal infection whilst 33% of wild-type mice survived (Figure 6A). Plating of aliquots of blood obtained from the tail veins of mice 48 h after i.n. inoculation demonstrated that all the *Apcs^−/−^* mice had large numbers of bacteria in the blood, whereas only 44% of wild-type mice had detectable septicaemia (Figure 6B). Hence, *Apcs^−/−^* mice are more susceptible to S. pneumoniae pneumonia, and this increased susceptibility is associated with increased levels of bacteria infection in the blood.

### SAP Aids Control of S. pneumoniae Replication in the Lung and Blood

To characterise the role of SAP during innate immunity in more detail, groups of wild-type and *Apcs^−/−^* mice were culled 4 and 24 h after i.n. inoculation with 1 × 10^6^ cfu of D39 and the number of bacteria present in target organs calculated by plating serial dilutions of bronchoalveolar fluid (BALF), lung and spleen homogenates, and the blood. After 4 h of infection, there were slightly higher levels of S. pneumoniae cfu in BALF and lung from *Apcs^−/−^* mice compared to wild-type mice (Table 1). By 24 h after inoculation, *Apcs^−/−^* mice had over 1 log greater bacterial cfu in the BALF, 2 logs in lung homogenates, and 4 logs in the blood compared to wild-type mice (Table 1), demonstrating that *Apcs^−/−^* mice were unable to control bacterial replication within the lung and systemic circulation. To ensure that chromosomal translocation of 129 DNA surrounding the SAP gene into the C57BL/6 mouse background was not responsible for the increased susceptibility of the *Apcs^−/−^* mice to S. pneumoniae infection, experiments were repeated using the C57BL/6.129(D1Mit105–223) congenic mice and C57BL/6 animals. No differences in bacterial cfu were identified between these strains 24 h after inoculation of ST2 S. pneumoniae, indicating that SAP deficiency is likely to be responsible for the phenotype seen in *Apcs^−/−^* mice (unpublished data). To further link the observed increased susceptibility of *Apcs^−/−^* mice to S. pneumoniae pneumonia to deficiency in SAP, *Apcs^−/−^* mice were supplemented by tail vein injection with 5 mg/kg of hSAP 1 h prior to inoculation with D39, and the bacterial cfu in target organs obtained at 24 h. Although there was wide variation in the numbers of bacteria recovered between mice, for all target organs the median S. pneumoniae cfu recovered from *Apcs^−/−^* mice complemented with hSAP were similar to those for wild-type mice and 1 to 4 log fewer than the median cfu recovered from *Apcs^−/−^* mice given PBS alone (Table 1).

**Table 1:**
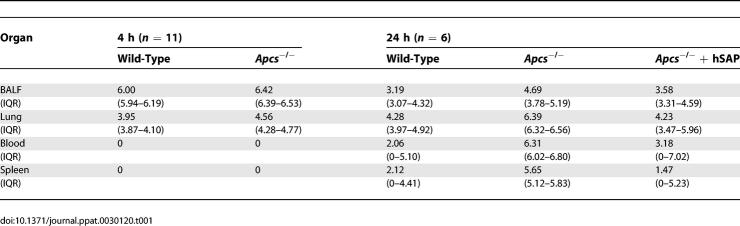
Median cfu/ml Recovered from Target Organs of Wild-Type and *Apcs* ^−/−^ Mice (with and without Supplementation with 5 mg/kg hSAP Intravenously) after i.n. Inoculation of 1 × 10^6^ cfu of S. pneumoniae ST2 Strain D39

### Inflammatory Responses to S. pneumoniae Pneumonia in *Apcs^−/−^* Mice

**Figure 7:**
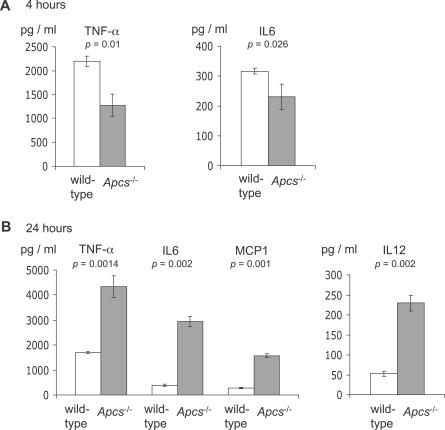
Cytokine Levels in BALF Recovered from Wild-Type or *Apcs* ^−/−^ Mice 4 h or 24 h after i.n. Inoculation with 1.0 × 10^6^ cfu of ST2 S. pneumoniae Error bars represent SDs, and the *p*-values (2-tailed *t* tests) for comparison between wild-type and *Apcs* ^−/−^ mice are given below the title for each panel. (A) Cytokine levels 4 h after inoculation. (B) Cytokine levels 24 h after inoculation.

As well as opsonising bacteria, activation of the complement system stimulates pro-inflammatory responses to infection. To investigate the effect of SAP deficiency on the inflammatory response to S. pneumoniae pneumonia, the levels of pro-inflammatory cytokines were measured in BALF from wild-type and *Apcs^−/−^* mice 4 h and 24 h after i.n. inoculation with 1 × 10^6^ cfu of the S. pneumoniae D39 strain. At 4 h after inoculation, despite the slightly higher numbers of bacterial cfu in BALF and lungs of *Apcs^−/−^* mice (Table 1), the levels of TNF-α and IL-6 were lower in *Apcs^−/−^* mice than in wild-type mice (Figure 7A), suggesting that at this early stage of infection *Apcs^−/−^* mice had an impaired inflammatory cytokine response to S. pneumoniae pneumonia. Levels of IL-12, IL-10, MCP-1, or IFN-γ in BALF at 4 h were very low or undetectable (unpublished data). By 24 h, at which time point *Apcs^−/−^* mice had considerably greater numbers of S. pneumoniae within target organs (Table 1), the levels of IL-6, IL-12, TNF-α, and MCP-1 in BALF were raised in *Apcs^−/−^* mice compared to those of wild-type mice (Figure 7B). The levels of IL-10 and IFN-γ in BALF at 24 h remained very low (unpublished data). The consequences of differences in inflammatory cytokines between *Apcs^−/−^* and wild-type mice were assessed by scoring the level of inflammation in lung sections. There were no significant differences in the score for the degree of histological inflammation of the lungs 4 h after inoculation (a median score of 15 for both *Apcs^−/−^* and wild-type mice), and although there was an increase in the inflammation score 24 h after infection in the lungs from *Apcs^−/−^* mice, with a median score of 70 (IQR 60–80) compared to a median score of 30 (IQR 19–68) for wild-type mice, this did not reach statistical significance (*p =* 0.48). Overall, these results suggest that during the early stages of S. pneumoniae pneumonia, there is a more pronounced pro-inflammatory response in wild-type mice compared to *Apcs^−/−^* mice. However, at later stages of infection, when there is considerably greater bacterial cfu in the target organs of *Apcs^−/−^* mice (Table 1), *Apcs^−/−^* mice have more pronounced levels of inflammation than wild-type mice.

## Discussion

The pentraxin SAP is an abundant plasma protein in both humans and mice, but its physiological role is not fully understood. By analogy to the related proteins CRP, which is known to mediate complement-dependent immunity [38,39,45], and Pentraxin3 [46], a role for SAP in innate immunity has been suggested. This possibility is supported by data demonstrating that SAP can bind to pathogen-associated structures such as PC and LPS [16,17,20]. Furthermore, SAP may interact with the classical pathway component C1q through its collagen binding site, and possibly stimulates phagocytosis through Fcγ receptors [9,10,13,14]. However, the physiological relevance of these observations is unclear, and although in vitro phenotypes associated with hSAP suggest it may protect against a variety of pathogens, including tuberculosis, malaria, or influenza A [22,25–27], other authors have shown that in mice SAP actually aids the virulence of S. pyogenes and *E. coli*, possibly by preventing classical pathway–mediated complement activity and phagocytosis [29,30].

Using SAP-deficient mice, we have investigated the biological role of SAP during infection by the Gram-positive pathogen *S. pneumoniae.* We have previously shown that the classical pathway is vital for innate immunity to S. pneumoniae, partially through recognition of S. pneumoniae by natural IgM [34]. However, natural IgM-deficient mice were markedly less susceptible to S. pneumoniae infection than C1q-deficient mice [34], suggesting there are other mediators of classical pathway activity against S. pneumoniae. The lectin SIGN-R1 has recently been shown to be one such mediator, with binding of the S. pneumoniae capsule to SIGN-R1 resulting in activation of the classical pathway [41]. However, although SIGN-R1-deficient mice have an increased susceptibility to S. pneumoniae infection, like natural IgM mice they are more resistant than C1q-deficient mice [41], indicating that additional molecules may contribute to complement activation. CRP is also thought to bind to S. pneumoniae and activate the classical pathway [40,47], but is present only in low levels in mice and therefore probably does not contribute strongly to innate immunity in the mouse models of S. pneumoniae infection. As SAP from different mammalian species can bind both to PC and C1q [9,10,16,17], we hypothesised that SAP could also contribute to the activation of the classical pathway by S. pneumoniae, and therefore aid both innate and acquired immunity to this important pathogen. This hypothesis is supported by our data showing binding of hSAP to three different capsular serotypes of S. pneumoniae, and impaired C3b deposition on these S. pneumoniae strains in *Apcs^−/−^* serum compared to serum from wild-type mice. Furthermore, we have shown that the binding of human C1q to S. pneumoniae is increased by the presence of hSAP and that the effects of SAP on complement are dependent on an intact classical pathway. The reduced complement activity in SAP-deficient serum versus S. pneumoniae results in an impaired ability of SAP-deficient mice to control *S. pneumoniae* replication within both the lungs and the bloodstream, leading to uncontrolled infection in a mouse model of pneumonia. This phenotype is very similar to that seen in mice deficient in natural IgM [34], and the data support the hypothesis that SAP and natural IgM both contribute, along with SIGN-R1 and CRP, towards classical pathway–mediated immunity to S. pneumoniae. Previous reports that SAP does not aid immunity to S. pneumoniae after intravenous (i.v.) inoculation [48] used wild-type mice infected with bacteria that had been incubated with SAP rather than SAP-deficient animals, and this model was therefore probably too insensitive to identify the effects we have shown for SAP.

Reduced complement activity results in increased susceptibility to infection by impairing C3b-mediated clearance of bacteria by phagocytes and/or by decreasing complement-mediated inflammatory responses to infection [44,49,50]. Our results suggest that both mechanisms could affect the susceptibility of *Apcs^−/−^* mice to S. pneumoniae. Uptake of the three different S. pneumoniae strains by a neutrophil-like cell line was impaired in *Apcs^−/−^* serum, and clearance of S. pneumoniae from the systemic circulation after i.v. inoculation, which is mainly dependent on phagocytosis by the reticuloendothelial system [51], was markedly reduced in *Apcs^−/−^* mice. These data suggest that by reducing opsonisation of S. pneumoniae with C3b, SAP deficiency results in impaired phagocytosis. The effects of SAP on phagocytosis of S. pneumoniae were serum- and classical pathway–dependent, with no stimulation of phagocytosis when SAP was added to medium alone or serum deficient in C1q, indicating that SAP assisted phagocytosis through classical pathway activity and not by direct binding to Fcγ receptors. In addition, we found that in BALF from *Apcs^−/−^* mice obtained at an early stage of infection there were lower levels of the pro-inflammatory cytokines TNF-α and IL-6 despite containing slightly greater numbers of cfu than wild-type mice in BALF and lung homogenates at this stage. Hence, the early pro-inflammatory response to S. pneumoniae pneumonia was impaired in *Apcs^−/−^* mice, and this may contribute to the increased susceptibility of these mice. Whether the reduced inflammatory responses in *Apcs^−/−^* mice are due to loss of direct effects of SAP on modulating the inflammatory response, or is secondary to reduced complement activation and phagocytosis, requires further evaluation. The increased inflammation in *Apcs^−/−^* mice compared to wild-type mice at 24 h probably reflects the overwhelming infection present in *Apcs^−/−^* mice at this stage rather than direct effects of SAP deficiency.

Although human and murine SAP have a high degree of homology at the amino acid level and both bind to PC, they do have some differences in their structure and interactions with other proteins [19]. Furthermore, CRP is the major component of the acute phase response in humans and SAP, although present in high concentrations in human sera, is the major acute phase response protein in mice [2]. Hence, to identify the possible human relevance of results obtained with *Apcs^−/−^* mice, we have complemented our assays using hSAP. Complementation of *Apcs^−/−^* serum with hSAP restored C3b deposition on bacteria and phagocytosis close to the levels seen with wild-type serum, and administration of hSAP before infection with S. pneumoniae increased the resistance of *Apcs^−/−^* mice to infection. Furthermore, the evidence for SAP-dependent C1q deposition on S. pneumoniae was obtained with human reagents. These data suggest that SAP is also important for classical pathway–mediated host immunity to S. pneumoniae in humans as well as mice. As S. pneumoniae is one of the commonest causes of infant mortality in the developing world [32], a role for SAP in preventing serious S. pneumoniae infections helps explain why there is no natural state of SAP deficiency.

In contrast to our results with S. pneumoniae pneumonia, *Apcs^−/−^* mice are protected against infection with S. pyogenes inoculated intraperitoneally despite a high level of SAP binding to the bacteria [30]. A possible explanation for the differences in the effect of SAP on immunity between these related pathogens could be differences in their interaction with the complement system. We have previously shown that the classical pathway is the dominant pathway for innate immunity to S. pneumoniae [34], whereas the alternative pathway is more important for innate immunity to S. pyogenes [52]. As the classical pathway does not contribute strongly towards complement activation by most strains of S. pyogenes [52], SAP may not be able to aid innate immunity to this pathogen. In addition, S. pyogenes does not express PC on its surface, and to which bacterial surface structure SAP binds may influence its functional role and interactions with complement factors. For example, de Haas et al. have reported that in direct contrast to the results presented in this manuscript, binding of SAP to the LPS expressed by some E. coli strains inhibits classical pathway–mediated complement activity, perhaps by preventing direct binding of C1q to LPS [29]. Further research is required to identify whether SAP mediates complement-dependent immunity to other important pathogens, and to determine why SAP has contrasting effects on susceptibility to closely related pathogens such as S. pneumoniae and S. pyogenes.

In summary, we have demonstrated that SAP aids complement activity against S. pneumoniae and is an important component of the innate immune response to this pathogen. To our knowledge, this is the first report demonstrating a positive role for SAP in complement-mediated immunity to a microbial pathogen. The data make a significant contribution to our understanding of the biological role of SAP and to our knowledge of the complex mechanisms leading to activation of complement by S. pneumoniae.

## Materials and Methods

### Bacteria.

S. pneumoniae strains belonging to capsular STs 2 (D39), 4 (JSB4, previously M313), and 23F (JSB23F, previously Io11697) were used for the majority of the studies [53]. The E. coli strain O111:B4 and the S. pyogenes strain H372 were used for SAP binding assays [30,52]. S. pneumoniae and S. pyogenes strains were cultured in Todd-Hewitt broth supplemented with 0.5% yeast extract (Oxoid, http://www.oxoid.com/) while E. coli O111:B4 was cultured in LB broth (Oxoid). All strains were grown to an optical density (OD_580_) of 0.4 (corresponding to about 10^8^ cfu/ml) and stored at −70 °C in 10% glycerol as single-use aliquots.

### ELISA for SAP binding to S. pneumoniae.

SAP binding to S. pneumoniae was analysed by whole-cell ELISA as previously described [54]. Briefly, bacterial cultures from late log phase were resuspended in PBS to an OD_550_ of 1.0, 200 μl of this suspension added to each well of 96-well plates (Nunc MaxiSorp, http://www.nuncbrand.com/), air dried at room temperature, and blocked with 200 μl of PBS-0.5 % BSA-NaN_3_ for 1 h before 50 μl of different concentrations of hSAP (Calbiochem, http://www.emdbiosciences.com/html/CBC/home.html) were added to each well. After incubation overnight at 4 °C, the plates were incubated with 50 μl of rabbit anti-human SAP (Calbiochem) diluted 1/2000 for 5 h at 4 °C, incubated overnight with 50 μl of goat anti-rabbit AP (Sigma, http://www.sigmaaldrich.com/) diluted 1/1000, and developed using FAST p-nitrophenyl phosphate (Sigma) for 30 min before determining the OD_405_ using a microtiter plate reader (Multiskam ACC/340; Titertek, http://www.titertek.com/).

### C3b, C1q, CRP, and SAP Binding to S. pneumoniae.

C3b deposition on S. pneumoniae was measured using a flow cytometry assay as previously described [34,52]. Briefly, 10^7^ cfu of S. pneumoniae were incubated with 10 μl of serum from wild-type or *Apcs^−/−^* mice and bacteria coated with C3b identified using a FITC-goat anti mouse C3 antibody and flow cytometry. CRP, SAP, and C1q binding assays were performed by a similar assay using either rabbit anti-human SAP or CRP (Calbiochem, with an appropriate FITC labelled secondary antibody) or FITC sheep anti-human C1q antibody (Serotec, http://www.ab-direct.com/), and incubating at 37 °C S. pneumoniae for 1 h with serum with or without addition of EDTA or PC (Sigma), 10 or 50 μg/ml hSAP (Calbiochem), and/or human C1q protein (Calbiochem). C3 levels (measured by ELISA) in the serum of *Apcs^−/−^* and wild-type mice were similar at 332 mg/l SD 101.7 (*n* = 40) for *Apcs^−/−^* mice, and 357 mg/l SD 123.5 (*n* = 20) for C57B/6 mice. Serum deficient in both C1q and SAP was obtained from *Apcs^−/−^.C1qa^−/−^* mice created by interbreeding the previously described *Apcs^−/−^* and *C1qa^−/−^* mouse strains [5,55]. C1q binding assays were also performed in human serum depleted in C1q (Calbiochem) with or without addition of 50 μg/ml of hSAP protein, using a serum that had been treated similarly by depletion of a terminal complement pathway component (C9, Calbiochem) to represent normal serum.

### Phagocytosis assays.

Phagocytosis of S. pneumoniae in serum from *Apcs^−/−^*, * Apcs^−/−^.C1qa^−/−^*, and wild-type mice was investigated using a previously described flow cytometry assay and the human tissue culture cell line HL-60 (promyelocytic leukemia cells; CCL240; American Type Culture Collection, http://www.atcc.org/) differentiated into granulocytes [53,56]. S. pneumoniae were fluorescently labelled with 5,6-carboxyfluorescein succinimidyl ester (FAM-SE; Molecular Probes, http://probes.invitrogen.com/) as described and stored at −70 °C in 10% glycerol as single-use aliquots (7 × 10^8^ cfu/ml). FAM-SE-labelled bacteria (10^6^ cfu) were opsonised with 10 μl of dilutions of serum obtained from wild-type or *Apcs^−/−^* mice in a 96-well plate for 20 min at 37 °C with horizontal shaking (150 rpm). HL-60 cells (10^5^) were added to each well and incubated for 30 min at 37 °C, fixed with 3% PFA, and analysed using a FACScalibur flow cytometer (minimum of 6,000 cells per sample) to identify the proportion of cells associated with fluorescent bacteria as a marker of phagocytosis [43].

### Infection experiments.

Wild-type C57BL/6 mice were purchased from commercial breeders, and *Apcs^−/−^* and C57BL/6.129(D1Mit105–223) congenic mice were bred in-house by one of the authors (MB) [5,42]. All mice used were 8–16 wk old, and within each experiment groups of mice were matched for age and sex. Studies were performed according to UK Home Office and university guidelines for animal use and care. Mice were inoculated i.n. (under halothane anesthesia, 1 × 10^6^ cfu/mouse) or intravenously (1 × 10^6^ cfu/mouse) with the S. pneumoniae D39 strain appropriately diluted in PBS. For survival studies, mice were killed when they exhibited signs of severe disease from which recovery was unlikely [57]. For experiments to test the number of cfu in different target organs or to perform immunological analysis, target organs were recovered 4 and 24 h after inoculation as previously described [34]. Bacterial counts were calculated by plating serial dilutions of the homogenised organs suspensions, blood, and BALF onto blood agar and incubated at 37 °C in 5% CO_2_.

### Cytokine levels and histological analysis of inflammation.

The levels of inflammatory cytokines and chemokines (IL-6, IL-10, MCP-1, IFN-γ, TNF-α, and IL-12p70) in BALF were analysed by flow cytometry in 50 μl of pooled BALF from wild-type or *Apcs^−/−^* mice using the Mouse Inflammation Cytometric Bead Array kit (Becton Dickinson, http://www.bdbiosciences.com/) according to manufacturer protocols and using the BD CBA software [52]. For the histological analysis of inflammation, in a proportion of infection experiments the left lung was fixed in 4% neutral buffered formalin, processed to paraffin wax, and stained with haematoxylin and eosin. Inflammation was assessed using a simplified score based on a previously described scoring system for inflammation during S. pneumoniae pneumonia [58]. The extent of lung involvement was estimated by examining lung cross sections at ×10 magnification. The degree of inflammation for six fields was scored at ×200 magnification as 1 (no visible inflammatory change), 2 (minimal swelling of alveolar walls with slight change in architecture), 3 (increased swelling with presence of erythrocytes and inflammatory cells and an increase in type II pneumocytes), and 4 (considerable haemorrhage with inflammatory cell influx, widespread alveolar disorganisation with interstitial swelling and pneumocyte proliferation). A total score for each mouse was obtained by multiplying the percentage of involved lung by the mean score for the areas analysed, and data presented as medians with IQRs.

### Statistical analysis.

The complement factor binding and opsonophagocytosis data presented are representative of results obtained from several independent experiments. The data for mouse infection experiments are representative of duplicate experiments that gave similar results. The results of C3b deposition, C1q, CRP and SAP binding experiments, cytokine levels, and phagocytosis assays were analysed using 2-tailed *t* tests. Bacterial cfu recovered from target organs and histology scoring were analysed using the Mann–Whitney *U* test for non-parametric data. Differences in survival curves between mouse strains were compared using the log rank method. All error bars given on the figures represent standard deviations.

## Abbreviations

BALF: bronchoalveolar fluid
CRP: C reactive protein
HBSS: Hank's Balanced Salt Solution
IQR: interquartile range
i.v.: intravenous
i.n.: intranasal
LPS: lipopolysaccharide
PC: phosphorylcholine
SAP: serum amyloid P
SD: standard deviation
ST: serotype
